# Supplementary material for: Oral Administration of Rhamnan Sulfate from Monostroma nitidum Suppresses Atherosclerosis in ApoE-Deficient Mice Fed a High-Fat Diet
Source: Cells. 2023 Nov 20;12(22):2666. doi: 10.3390/cells12222666 (PMC10670814; doi:10.3390/cells12222666)
Supplement: Supplementary file 1 [file cells-12-02666-s001.zip › cells-2686024-supplementary.pdf]

Supplementary Material for

Oral Administration of Rhamnan Sulfate from *Monostroma nitidum* Suppresses Atherosclerosis in ApoE-Deficient Mice Fed a High-Fat Diet

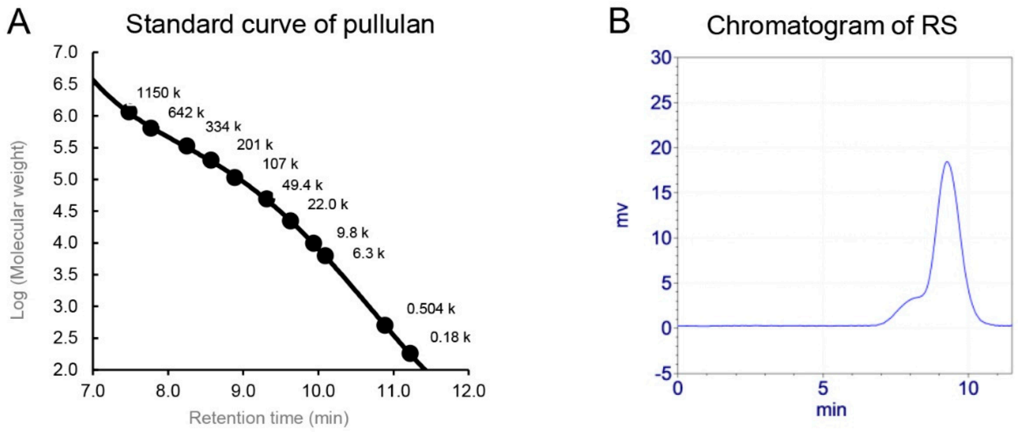

Figure S1. Characteristics of the rhamnan sulfate (RS) purified from *Monostroma nitidum*. (A) Standard curve of pullulan with different molecular weights (0.18, 0.50, 6.3, 9.8, 22.0, 49.4, 107, 201, 334, 642, 1150 kDa); (B) Chromatogram of purified RS by gel permeation chromatography (GPC) using a Shodex SB-806M HQ column and a Shodex RI-71 refractive index detector.

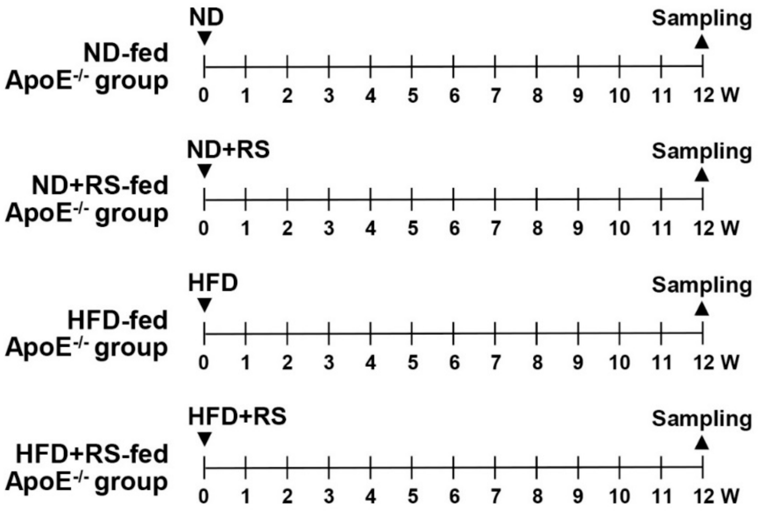

Figure S2. Animal diet and experimental schedule. Apolipoprotein E-deficient ($ApoE^{-/-}$) female mice were fed either a normal diet (ND), a normal diet containing 0.1% RS (ND+RS), a high-fat diet (HFD), or a high-fat diet containing 0.1% RS (HFD+RS) for 12 weeks. Thereafter, samples (plasma, liver, and abdominal aorta) were obtained from each mouse and used for experiments.

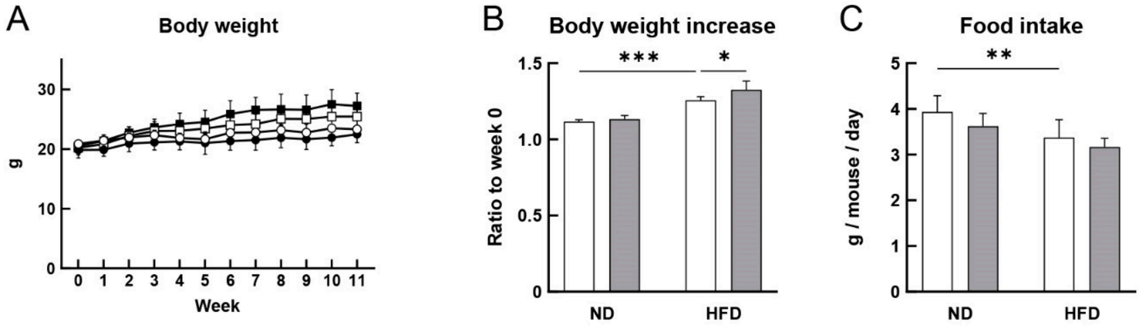

Figure S3. Effect of RS on body weight change and food intake (A) Changes in the body weight of ApoE$^{-/-}$ mice fed ND (open circles), ND+RS (closed circles), HFD (open squares), or HFD+RS (closed squares), respectively. (B) Rate of increase of the body weight of ApoE$^{-/-}$ mice fed ND (white column), ND+RS (gray column), HFD (white column), or HFD+RS (gray column) for 12 weeks compared to body weights before the start of the experiment. (C) Average daily dietary intake of ApoE$^{-/-}$ mice fed with ND (white column), ND+RS gray column), HFD (white column), or HFD+RS (gray column), for 12 weeks. The data are shown as mean ± SD. *$p < 0.05$, **$p < 0.01$, and ***$p < 0.001$ show significant differences between the respective groups by two-way analysis of variance (ANOVA, n = 6).

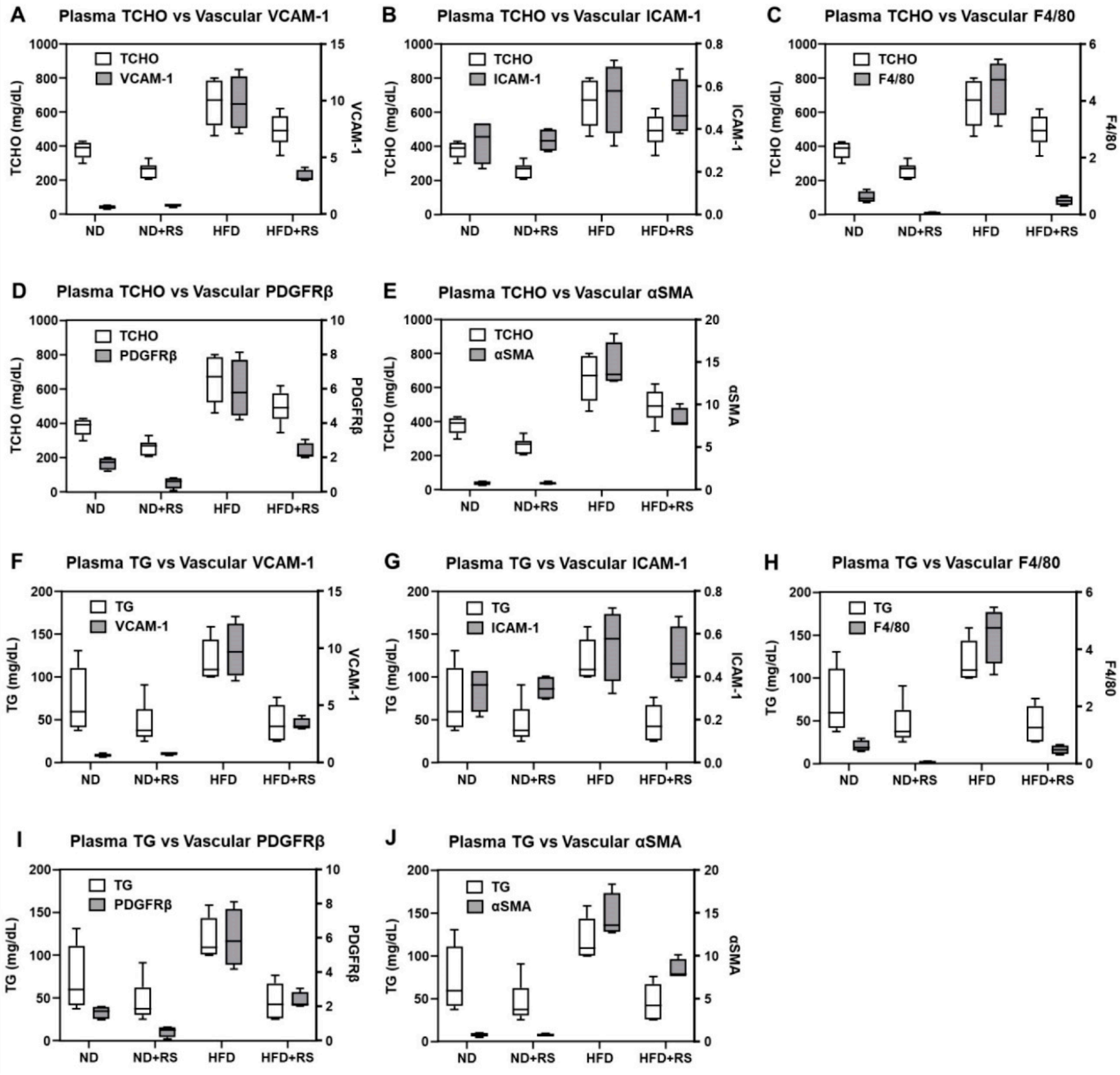

Figure S4. The relationship between plasma total cholesterol (TCHO) or triglyceride (TG) levels and expression levels of aortic molecules in ND- or HFD-fed ApoE$^{-/-}$ mice on ND or HFD with or without RS. A-E show the relationship between plasma TCHO levels and expression levels of vascular cell adhesion molecule-1 (VCAM-1), intercellular adhesion molecule 1 (ICAM-1), F4/80, platelet-derived growth factor receptor β (PDGFRβ), and α smooth muscle actin (αSMA), respectively. F-J show the relationship between plasma TG levels and expression levels of VCAM-1, ICAM-1, F4/80, PDGFRβ, and αSMA, respectively.

Table S1. Primer sequences used to determine mRNA expression levels of factors associated with inflammation and atherosclerosis in mice for qPCR

**Gene**	**Forward primer**	**Reverse primer**	**Product size (bp)**
Hprt	ATGGACTGATTATGGACAGGACTG	TCCAGCAGGTCAGCAAAGAAC	124
Mmp2	AACGGTCGGGAATACAGCAG	GTAAACAAGGCTTCATGGGGG	125
Mmp9	CGTCGTGATCCCCACTTACT	AACACACAGGGTTTGCCTTC	225
Srebf1	TCCAGTGGCAAAGGAGGCAC	CAGCATGCTCATTCGCTGCC	138
Icam1	GGTTCTCTGCTCCTCCACAT	CCTTCCAGGCTTTCTCTTTG	158
Vcam1	ACTCCCGTCATTGAGGATATTG	TGACAGTCTCCCTTTCTTTGAG	97

Hprt: hypoxanthine phosphoribosyltransferase 1, *Mmp2*: matrix metallopeptidase 2, *Mmp9*: matrix metallopeptidase 9, *Srebf1*: sterol regulatory element binding transcription factor 1, *Icam1*: intercellular adhesion molecule 1, *Vcam1*: vascular cell adhesion molecule 1.
